# Impact of Electronic Patient-Reported Outcomes on Unplanned Consultations and Hospitalizations in Patients With Cancer Undergoing Systemic Therapy: Results of a Patient-Reported Outcome Study Compared With Matched Retrospective Data

**DOI:** 10.2196/55917

**Published:** 2024-05-06

**Authors:** Andreas Trojan, Christian Kühne, Michael Kiessling, Johannes Schumacher, Stefan Dröse, Christian Singer, Christian Jackisch, Christoph Thomssen, Gerd A Kullak-Ublick

**Affiliations:** 1 Oncology Breast Center Zürichsee Horgen Switzerland; 2 Department of Clinical Pharmacology and Toxicology University Hospital Zurich University of Zurich Zurich Switzerland; 3 palleos healthcare Wiesbaden Germany; 4 Center for Breast Health and Female Medicine University Hospital Vienna Vienna Austria; 5 Sana Clinic Offenbach Offenbach Germany; 6 Evangelische Kliniken Essen-Mitte GmbH Essen Germany; 7 Department of Gynecology Martin Luther University Halle-Wittenberg Halle Germany

**Keywords:** systemic cancer therapy, electronic patient-reported outcome, ePRO, ePROs, Consilium Care, medidux, unplanned consultation, hospitalization, hospitalizations, hospitalized, cancer, oncology, side effect, side effects, adverse, chemotherapy, patient reported outcome, PRO, PROs, mobile health, mHealth, app, apps, application, applications, mobile phone

## Abstract

**Background:**

The evaluation of electronic patient-reported outcomes (ePROs) is increasingly being used in clinical studies of patients with cancer and enables structured and standardized data collection in patients’ everyday lives. So far, few studies or analyses have focused on the medical benefit of ePROs for patients.

**Objective:**

The current exploratory analysis aimed to obtain an initial indication of whether the use of the Consilium Care app (recently renamed medidux; mobile Health AG) for structured and regular self-assessment of side effects by ePROs had a recognizable effect on incidences of unplanned consultations and hospitalizations of patients with cancer compared to a control group in a real-world care setting without app use. To analyze this, the incidences of unplanned consultations and hospitalizations of patients with cancer using the Consilium Care app that were recorded by the treating physicians as part of the patient reported outcome (PRO) study were compared retrospectively to corresponding data from a comparable population of patients with cancer collected at 2 Swiss oncology centers during standard-of-care treatment.

**Methods:**

Patients with cancer in the PRO study (178 included in this analysis) receiving systemic therapy in a neoadjuvant or noncurative setting performed a self-assessment of side effects via the Consilium Care app over an observational period of 90 days. In this period, unplanned (emergency) consultations and hospitalizations were documented by the participating physicians. The incidence of these events was compared with retrospective data obtained from 2 Swiss tumor centers for a matched cohort of patients with cancer.

**Results:**

Both patient groups were comparable in terms of age and gender ratio, as well as the distribution of cancer entities and Joint Committee on Cancer stages. In total, 139 patients from each group were treated with chemotherapy and 39 with other therapies. Looking at all patients, no significant difference in events per patient was found between the Consilium group and the control group (odds ratio 0.742, 90% CI 0.455-1.206). However, a multivariate regression model revealed that the interaction term between the Consilium group and the factor “chemotherapy” was significant at the 5% level (*P*=.048). This motivated a corresponding subgroup analysis that indicated a relevant reduction of the risk for the intervention group in the subgroup of patients who underwent chemotherapy. The corresponding odds ratio of 0.53, 90% CI 0.288-0.957 is equivalent to a halving of the risk for patients in the Consilium group and suggests a clinically relevant effect that is significant at a 2-sided 10% level (*P*=.08, Fisher exact test).

**Conclusions:**

A comparison of unplanned consultations and hospitalizations from the PRO study with retrospective data from a comparable cohort of patients with cancer suggests a positive effect of regular app-based ePROs for patients receiving chemotherapy. These data are to be verified in the ongoing randomized PRO2 study (registered on ClinicalTrials.gov; NCT05425550).

**Trial Registration:**

ClinicalTrials.gov NCT03578731; https://www.clinicaltrials.gov/ct2/show/NCT03578731

**International Registered Report Identifier (IRRID):**

RR2-10.2196/29271

## Introduction

An important component of optimal medical care is considered to be guaranteed when patients actively participate in their treatment and are involved in decisions about their treatment plan [1]. The use of smartphones during the outpatient treatment of patients with cancer can enable low-threshold contact between patients and their treatment centers and facilitate patient involvement. The data recorded by patients (patient reported outcomes; PROs) can be recorded electronically in real time (electronic patient reported outcomes; ePROs) using a smartphone app and made available to the doctor. The use of ePROs thus enables ongoing recording of patients’ daily well-being and state of health. The evaluation of ePROs is also used in clinical studies of patients with cancer [2] and enables structured and standardized data collection in patients’ everyday lives. By analyzing the information flows and anonymized data of many patients, as well as by networking with other (research) centers, a basis can be created that can promote the quality and efficiency of treatment.

However, the benefits of app-based ePROs for patients can go beyond improving communication. It is known that patients with cancer undergoing systemic therapy often obtain side effects, with fatigue (80%), pain (48%), and nausea or vomiting (48%) being the most common [3]. The type of chemotherapy and the patient’s “performance index” are associated with the hospitalization rate [4]. It has been reported that 35% of newly diagnosed patients with cancer experienced an unplanned hospitalization, and 67% of hospitalized patients had previously been to the emergency department [5]. In 154 patients with colorectal cancer, 28% of hospitalizations were due to complications of cancer treatment, of which 19% were identified as potentially preventable [6]. Another study of patients with colorectal cancer showed that the majority of unplanned consultations (72%) occurred within 30 days of their last chemotherapy treatment. Of these unplanned visits, 10% resulted in hospitalization [7]. Among the unplanned hospital admissions of outpatients summarized in a retrospective study, 74% had received chemotherapy in the previous 6 months. Further, 69.7% of these hospitalizations occurred within 4 weeks of receiving chemotherapy. It can be assumed that by structured recording of patient reported symptoms, adverse events of cancer therapy can be recognized at an early stage and higher degrees of severity can be avoided through timely action. This is supported by recent studies. It was shown that the use of a digital app or web-based system for symptom monitoring had reduced the number of emergency admissions and hospitalizations compared to a control group and had even extended (progression-free) survival times [8-11]. A reduction in the number of serious adverse events (SAEs) compared to the control group could also be attributed to the use of the digital application in 2 studies [9,12].

Consilium Care (recently renamed medidux) is a digital app for monitoring and alleviating symptoms during and after the treatment of patients with cancer. The app enables the standardized entry of symptoms according to the Common Terminology Criteria for Adverse Events (CTCAE). When entering symptoms, users receive tips on how to alleviate them and are prompted to contact their treating physician or clinic if defined severity threshold values are exceeded. By sharing data with the treating physician, treatment can be customized precisely to the patient’s individual needs. The aim of Consilium Care or medidux is to improve the quality of life of patients with breast cancer, enable early symptom monitoring, and establish a closer connection with their treatment teams. In an initial study (registered on ClinicalTrials.gov; NCT02004496), it was shown that patient well-being and awareness of adverse effects could be improved by using the Consilium Care app in collaboration with the treating physician [13]. With further developed versions of the app, the benefits of digital patient monitoring using Consilium Care have also been demonstrated during immunotherapy and targeted therapies for cancer in the form of more efficient symptom assessment and patient-physician communication as well as a reduced need for telephone consultations [14-16]. In the PRO study (registered on ClinicalTrials.gov; NCT03578731), the primary end point investigated the extent to which the self-assessment of the severity of undesirable outcomes between patients with cancer using the Consilium Care app for 90 days and the treating physicians was consistent [17,18]. The occurrence of unplanned (emergency) consultations and hospitalizations among patients with cancer was recorded by the investigators as a secondary end point. As a control group without app usage was not included in the PRO study, retrospective patient data from a comparable cohort of patients with cancer were used for the present analysis to obtain initial indications as to whether a reduction in emergency consultations and hospitalizations can be observed when patients use the Consilium Care (medidux) app.

## Methods

### Data Collection of Unplanned Consultations and Hospitalizations Within the PRO Study’s Patient Population and Acquisition of Retrospective Data for the Control Group

For this retrospective comparison of the group of patients with cancer using the Consilium Care app with a control group that did not use the app, data were compiled from 2 different sources.

For the Consilium group, respective data were taken from the PRO study that was conducted as a multicenter, observational, noninterventional study. Patients with breast, colon, prostate, or lung cancer, as well as those with hematological malignancies, aged 18 years and older, receiving systemic therapy in a neoadjuvant or noncurative setting were eligible to participate after providing written informed consent. In addition, participants had to speak German and own a smartphone. Eligible participants were recruited consecutively and without preselection according to the recommendation of the local tumor boards in centers in Switzerland (10 recruited patients in Switzerland), Germany (2 centers), and Austria (1 center). The results corresponding to the primary objective (assessment of the level of agreement, κ, between symptom ratings by physicians at the time of the regular consultation and the ratings derived from the daily PRO between consultations) are published in Trojan et al [18]. Patients were assigned to medical oncology visits every 3 weeks and invited for shared reporting and intended symptom review, which were preferably scheduled on days of therapeutic intervention. The observation period covered a total of 90 days (for further details see Trojan et al [18]). Relevant to the present analysis is the recording of a secondary study end point by the participating physicians during the 3-weekly oncology visits: the number and reasons for unplanned consultations were surveyed and recorded, and unplanned (emergency) hospitalizations and their duration were documented. The latter were divided into ≤2 days and >2 days. In addition to the criteria defined in the analysis of the primary end point [18], only patients who completed the 90-day observation period according to the protocol were included in the present analysis, so that patients who died during the observation period or withdrew from this study for other reasons were not included. The group that used the app (Consilium group) finally consisted of 178 patients.

For the control group, which did not use the Consilium Care app, retrospective patient data were compiled from the databases of 2 oncology centers in Zurich. The patients were selected systematically in a 2-stage process whereby attention was initially only paid to a comparable composition of the patient collective concerning the most comparable distribution of therapy types, tumor entities, and tumor stages as well as age and gender. First, patients who had started and completed systemic cancer therapy between January 2016 and October 2020 were selected consecutively in reverse order to the completion date from the Zurich Oncology Center database, that is, starting with the patient who was the last to complete 90 days of cancer treatment and then continuing with the patient with the next completion date, etc. As the proportion of patients with breast cancer was still too small, a further 43 patient data sets from the Breast Center Zurich were selected also in reverse consecutive order. Patient data were recorded anonymously, with only the respective (pseudonymized) patient ID of the 2 centers serving as proof of identity during the compilation of the control group. The retrospective data of the selected patients were then searched for documented unplanned consultations and hospitalizations by a second person who was not involved in their selection. Patient demographics and relevant systemic cancer treatment data were also collected for a period of 90 days from the start of treatment.

### Ethical Considerations

The PRO study was approved by the responsible ethics committees in Switzerland (Lead EC: KEK-ZH: 2017-02028) Germany, and Austria and conducted per the principles of the Declaration of Helsinki and registered on ClinicalTrials.gov (NCT03578731). Patients with breast, colon, prostate, or lung cancer, as well as those with hematological malignancies, aged 18 years and older, and initiating adjuvant or neoadjuvant systemic therapy were eligible to participate after providing written informed consent. In addition, participants had to speak German and own a smartphone. Eligible participants were recruited consecutively and without preselection according to the recommendation of the local tumor boards in centers in Switzerland, Germany, and Austria.

All study documents were de-identified by assigning a unique ID to each patient. Functional data security was ensured by identification being made only possible via the patient’s ID. The data on the patient’s device were encapsulated in the app and the data exchange was encrypted with the patient’s ID. There was no compensation provided to participants.

Informed consents for the control group were present for the included patients of the 2 respective cancer centers. These were obtained as part of normal patient care based on the internal processes at the cancer centers. These generally provide a declaration of consent for patients upon admission, which allows for the anonymized use of collected data for research purposes. Patients can refuse consent without affecting further treatment and can also withdraw it at any time.

### Division of Patients Into Chemotherapy and Nonchemotherapy Subgroups

All participating patients in the PRO study as well as in the retrospective control group received systemic cancer therapy according to the local standard of care. Due to the large number of different systemic cancer therapies used as standard treatment for the cancer indications investigated, it was not possible to create a control group with exactly the same distribution of systemic drugs. Since the therapy forms’ influence on the number of unplanned consultations and hospitalizations was to be evaluated, the therapies were recorded, and the systemic therapies were divided into 2 groups: chemotherapy and nonchemotherapy. The chemotherapy group included therapies with classic chemotherapeutic agents such as alkaloids, alkylating agents, antitumor antibiotics, etc; the nonchemotherapy group included therapeutic agents such as antihormones, aromatase inhibitors, antibodies, checkpoint inhibitors, or cyclin-dependent kinase 4/6 inhibitors. If a patient’s treatment consisted of nonchemotherapeutic agents, such as antibodies, in addition to classic chemotherapeutic agents, the patient was assigned to the chemotherapy group according to the higher expected toxicity.

### Objective of Analysis

The objective of this retrospective analysis was to evaluate whether Consilium Care guided ePROs for symptom monitoring resulted in fewer unplanned consultations and hospitalizations (summarized as events) in patients with cancer compared to patients who did not use the app.

### Mobile App

In the PRO study, the Consilium Care app (version 2.0) was used. In brief, the app facilitated the selection of well-being, symptoms, medication, and private notes. Symptoms, which were structured in groups according to organ systems, could be selected. The symptom entry display (52 distinct symptoms were available for which severity, onset, and duration could be indicated) was equipped with date and time stamps. Symptom severity, with descriptions based on the CTCAE, could be selected via a slider. The symptom history was displayed on a timeline with individual colors for each symptom. In addition, diary entries and information on diagnosis and therapy were indicated separately. Patients were encouraged to capture data on well-being and symptoms daily. Recording usually started on the day of the therapy’s initiation or of a change in therapy and continued throughout an observational period of 90 days. The app allowed the continuous recording of well-being and symptoms based on the CTCAE through the use of virtual analogue scales. Information for self-care (derived from the Swiss Cancer League) was provided to them via the app depending on the severity of symptoms upon data entry. In the case of severe symptoms, patients were encouraged by push notifications to seek medical advice. The history of recorded data was displayed and visualized in the form of a symptom progression chart. For further information, refer to Trojan et al [18].

### Statistical Analyses

The question of whether the number of recorded unplanned consultations and hospitalizations per patient can be reduced by using the Consilium Care app was addressed by considering the incidence proportions (proportion of patients with at least one event) in both patient groups. Exploratory analysis was performed using the Fisher exact test and multivariate logistic regression. Effect sizes are reported as odds ratios (OR; Consilium group vs retrospective control group) with a 90% CI. The 2-sided 10% significance threshold was chosen to demonstrate initial trends in this exploratory setting that would warrant further investigation in a fully prospective trial. The results are reported for the overall group, as well as for the subgroup of patients who underwent chemotherapy. Analyses were performed with R (version 4.0.2; June 22, 2020; R Core Team).

## Results

### Baseline Characteristics

Both patient groups were comparable in terms of age (age range 23-83 years; mean age 54.2, SD 12.0 years in the Consilium group vs age range 27-85 years; mean age 56.4, SD 13.8 years in the control group), gender ratio (155 women and 23 men vs 157 women and 21 men), cancer entity (breast cancer n=139 vs n=133; lung cancer n=13 vs n=11, colorectal cancer n=11 vs n=15, hematological malignancies n=9 vs n=14, prostate cancer n=6 vs n=5) and American Joint Committee on Cancer stages (Table 1). Further, 139 patients from each group were treated with chemotherapy and 39 with other therapies (nonchemotherapy).

**Table 1:** Baseline characteristics.

Characteristics		Consilium group (n=178)	Control group (n=178)
**Age (years)**			
	Range	23-83	27-85
	Mean (SD)	54.2 (12.0)	56.4 (13.8)
**Sex** **, n (%)**			
	Female	155 (87)	157 (88)
	Male	23 (13)	21 (12)
**Cancer entity** **, n (%)**			
	Breast cancer	139 (78)	133 (75)
	Lung cancer	13 (7)	11 (6)
	Prostate cancer	6 (3)	5 (3)
	Colorectal cancer	11 (6)	15 (8)
	Hematological malignancies	9 (5)	14 (8)
**AJCC^a^ stage, n (%)**			
	Stage I	22 (12)	25 (14)
	Stage II	68 (38)	51 (29)
	Stage III	37 (21)	29 (16)
	Stage IV	51 (29)	73 (41)
**Treatment** **, n (%)**			
	Chemotherapy	139 (78)	139 (78)
	Nonchemotherapy	39 (22)	39 (22)

^a^AJCC: American Joint Committee on Cancer.

### Incidence of Unplanned Consultations and Hospitalizations

In the Consilium group, a total of 36 unplanned consultations and hospitalizations occurred during the observation period (Table 2). These 36 events occurred in 29 patients, with 1 event documented for 25 patients and 2 or 3 events for 2 patients each. The events could be divided into 23 unplanned consultations, 5 unplanned hospitalizations lasting up to 2 days, and 8 unplanned hospitalizations lasting longer than 2 days (Table 2). In comparison, there were a total of 38 documented unplanned consultations and hospitalizations in the control group, which were recorded in 37 patients. Further, 1 event was documented for 36 patients and 2 events for 1 patient. These were divided into 29 unplanned consultations, 4 hospitalizations up to a maximum of 2 days, and 5 unplanned hospitalizations of more than 2 days. Per the nominally largest proportion of patients with breast cancer in both groups, most events also occurred within this subgroup, with the remainder distributed among the 4 other cancer entities (Table 2).

Looking at the sheer numbers of events, no difference can be found between the Consilium and the retrospective control group, as already mentioned in Trojan et al [18]. Since multiple occurrences of events in a single patient cannot be assumed to be independent, the proportion of patients with at least one event (29 for the Consilium group and 37 for the control group) was used for the statistical analysis. Looking at all patients, there was also no statistically robust positive effect of app use on unplanned consultations and hospitalizations (OR 0.742, 90% CI 0.455-1.206; Fisher exact test *P*=.34 at a 2-sided 10% significance level).

To take into account the heterogeneity of the overall collective, a multivariate regression model was evaluated for the binary end point of the occurrence of at least one unplanned event (consultation or hospitalization). The model analyzed the factor of the intervention group (Consilium versus control group) and adjusted for treatment type and tumor entity. Due to the small subgroup sizes, the tumor entity variable was dichotomized into the values “breast cancer” and “other.” Furthermore, the model contains interaction terms between intervention and chemotherapy or breast cancer subgroups. Table 3 summarizes the results and reports the corresponding OR for all coefficients to interpret the effect size, as well as a *P* value, which indicates the statistical significance of the respective coefficient. Looking at the heterogeneous overall collective, no statistically robust positive effect of app use on unplanned consultations and hospitalizations can be detected in this model (Table 3; model term for Consilium group, *P*>.10). However, the interaction term between the Consilium group and the factor “chemotherapy” was significant at the 5% level (*P*=.048), indicating a relevant reduction of risk for the intervention group in the collective of patients who underwent chemotherapy. This motivated a further subgroup analysis within the collective of patients who underwent chemotherapy.

In each group, that is, the Consilium group and the control group, 139 patients had received chemotherapy. In the Consilium group, 17 (12.2%) patients with unplanned consultations and hospitalizations were recorded; in the control group, there were 29 (20.9%) patients. In the chemotherapy subgroup, a clinically relevant effect of app use on these events was observed, with an OR of 0.53, 90% CI 0.288-0.957 (Fisher exact test; *P*=.08) which is significant at a 2-sided 10% significance level. The observed OR is equivalent to a halving of the risk for patients in the intervention group.

**Table 2:** Occurrence of at least one event in patients.

Events		Consilium group (n=178)	Control group (n=178)
Patients with ≥1 event, n (%)		29 (16.3)	37 (20.8)
**Total events, n**		36	38
	Unplanned (emergency) consultations	23	29
	Hospitalizations ≤2 days	5	4
	Hospitalizations >2 days	8	5
**Subgroups for tumor stages,** **(events, n/patients, n)**			
	AJCC^a^ stage I	1/22	0/25
	AJCC stage II	9/68	9/51
	AJCC stage III	4/37	9/16
	AJCC stage IV	15/51	19/41
**Subgroups for therapies,** **(events, n/patients, n)**			
	Chemotherapy	17/139	29/139
	Nonchemotherapy	12/39	8/39
**Subgroups for cancer entities, n**			
	Breast cancer	22	23
	Lung cancer	2	4
	Prostate cancer	1	2
	Colorectal cancer	1	6
	Hematological malignancies	3	2

^a^AJCC: American Joint Committee on Cancer.

**Table 3:** Multivariate logistic regression for “unplanned consultation or hospitalization.”

Model term	Odds ratio (90% CI)	*P* value
Consilium group	1.16 (0.370-3.6220)	.83
Type of therapy: chemotherapy	1.09 (0.527-2.380)	.86
Cancer entity: breast cancer	0.46 (0.241-0.892)	.051^a^
Interaction Consilium group: chemotherapy	0.29 (0.101-0.805)	.048^b^
Interaction Consilium group: breast cancer	1.89 (0.681-5.455)	.31

^a^Above *P*<.05 significance.

^b^Below *P*<.05 significance.

## Discussion

### Principal Findings

In our comparative exploratory analysis of data from the PRO study and a matched retrospective control group, the effect of Consilium Care, an app for structured and regular self-assessment of side effects by ePROs, on unplanned consultations and hospitalizations of patients with cancer was analyzed. When considering all included patients, no statistically robust difference in unplanned consultations and hospitalizations between the groups could be demonstrated. In the Consilium group, there were a total of 36 unplanned consultations and hospitalizations, which occurred in 29 (16.3%) different patients. In comparison, the control group had 38 consultations and hospitalizations in 37 (20.8%) different patients. This simple comparison of the event numbers led Trojan et al [18] to conclude that a positive effect of the app was not demonstrable—but without showing the data and the analyses used. However, a multivariate regression model revealed that the interaction term between the Consilium group and the factor chemotherapy was significant at the 5% level (*P*=.048) and indicated a relevant reduction in the risk in the intervention group in the collective of patients who underwent chemotherapy. Within the subgroup of patients who underwent chemotherapy (139 in each group), 7 events were documented in the Consilium group, while 29 were recorded for the control group, which corresponds to a halving of the risk (OR 0.53, 90% CI 0.288-0.957) at a 2-sided 10% level. This indicates a relevant reduction in the risk in the Consilium group in the collective of patients who underwent chemotherapy and provides initial indications that the concomitant use of the Consilium Care app could have a positive, clinically relevant effect on patients with cancer receiving chemotherapy.

It should be noted that although care was taken to ensure a comparable composition of the 2 groups, they were still heterogeneous in terms of cancer entities, the American Joint Committee on Cancer stages, cancer therapies, and age structure. In addition, the data of the Consilium group in the PRO study were collected at 14 different centers—mainly in Switzerland, but also in Germany and Austria—while the retrospective data of the control group only came from 2 Swiss oncology centers. It should also be noted that within the PRO study, the participating physicians were explicitly asked in this study’s protocol to inquire about unplanned consultations and hospitalizations during standard patient visits. In contrast, the unplanned consultations and hospitalizations of the control group were drawn retrospectively from the patient records, which may have had a lower level of documentation in this respect. That a reduction in the events studied was only observed in the chemotherapy subgroup may be related to the fact that the majority of unplanned consultations and hospitalizations occur within 30 days of the last chemotherapy, a period that was covered by the chosen observation period [3,6]. Accordingly, any effects arising from app usage should have been able to manifest themselves during this period. Since the type of chemotherapy and the patient’s “performance index” are also associated with the hospitalization rate [3], it can be assumed that early detection of side effects supported by regular documentation of the patient in the form of app-based ePROs prevents higher severity and thus also reduces the hospitalization rate.

In other studies, with heterogeneous cancer populations, a positive clinically relevant effect of web- and smartphone-based apps that were used in a comparable way for monitoring and self-assessment of health status was demonstrated. Basch et al [7,8] were able to show that patients with metastatic solid tumors (metastatic breast, urogenital, gynecological, or lung cancers) receiving chemotherapy according to the standard of care were less likely to be admitted to the emergency department (34% vs 41% in the control group; *P*=.02) or hospitalized (45% vs 49%; *P*=.08) when regularly carrying out health self-assessment. In another study with patients with cancer (various tumor entities) who received approved oral cancer drugs, days of hospitalization (2.82 days vs 4.44 days, *P*=.02), and treatment-related toxicity ≥3 CTCAE (27.6% vs 36.9%, *P*=.02) were reduced in the Consilium group compared to a control group without the app [9]. The recently published results of the PreCycle study with a well-defined patient population (hormone receptor-positive, human epidermal growth factor receptor 2–negative locally advanced, or metastatic breast cancer) also show that not only was self-assessment-based quality of life greatly improved in a group of patients using a full version of a physician-supported app for regular self-assessment compared to a control group using an app with limited functionality [10], but also that patients using the full version were significantly less likely overall to have an SAE [11].

### Conclusions

The results of the analysis presented here clearly indicate a positive effect on the incidence of unplanned consultations and hospitalizations of an app that is used alongside cancer therapy to document side effects and support communication with the treating physician. This is also supported by studies with comparable apps, which also demonstrate a direct positive effect on the incidence of SAEs. Due to the exploratory nature of this study, the randomized PRO2 study (NCT05425550) is currently being conducted with the medidux app, the further-developed successor to the Consilium Care app, in which the incidence of high-grade adverse events (CTCAE >2) is being investigated in a better-defined patient population (patients with human epidermal growth factor receptor 2–positive breast cancer) under controlled conditions to support the results of this exploratory analysis.

## Abbreviations

CTCAE: Common Terminology Criteria for Adverse Events
ePRO: electronic patient-reported outcome
PRO: patient-reported outcome
OR: odds ratio
SAE: serious adverse event
